# Cold Water Bathing

**Published:** 1860-05

**Authors:** 


### COLD WATER BATHING.

Dr. is exhibiting the dimensions of his mental caliber, by furnishing the newspapers with the fact that he bathes in the river daily throughout the winter; usually runs two miles, plunges in, splurges about, and runs home. Rather think he hasn't much "practice" to attend to beyond that on his own person! He has sometimes to cut the ice, and takes his bath when the thermometer has been fifteen degrees below zero. Suppose it was a thousand; the water itself is no colder than if it were thirty-two degrees above. He frequently stands in the snow while using flesh-brush and towels; and dries himself by a cold north-east wind. Well! what is the advantage of this particular fuss every day? Why, that he has a good appetite, sleeps soundly, seldom takes cold, and never had disease of any kind. Rather an unfortunate confession! for a person seldom has but one disease in the body at a time: if he has gout, he has nothing else; if he has sick headache, he has nothing else; if cancer or consumption, nothing else. Again: there is a malady, a very serious one, whose existence all see and know and admit, except the unfortunate patient; and although it is daily wearing him to the grave, he can not be made to acknowledge its presence, and dies, believing himself a sound man. It is a disease of the upper story. For fear the reader may not "comprenez-vous," we will explain. When a man is a fool, you can't make him believe it; he will not medicate his malady; hence with all his experiences, he gets to be a bigger fool every day to the very last. The tendency of the article is to make persons believe that such heroic bathing prevents coughs, colds, and sickness in general.

Per contra: The Editor of this Journal has good health, sleeps soundly, seldom takes cold, has not swallowed a dose of medicine in many years, nor lost a meal for want of an appetite, always eats as much as he pleases, and never bathes in cold water. The last bath of that sort was nearly twenty years ago, on a Christmas-day, by jumping off the bow of a ship, into the Gulf of Mexico, "for the fun of the thing." He always washes face and hands in quite warm water, when it can be had; and the body once in a while. He drinks tea, coffee, etc. daily, and is about as spry in thought, word, and deed as most people. He might as well say that these "advantages" were owing to his washing in warm water, as the other doctor, that his immunity from coughs, colds, and sickness, was owing to his bathing in the river when the thermometer was below zero; or that avoiding liquors, tea, coffee, and tobacco made him a healthy man.

The probability is, that both of us had good health to begin with, and learned by regularity, carefulness, and temperance to take care of it. The "Propter hoc" mode of argument is exceedingly fallacious, the inference because a healthy old man did this, that, or the other thing all his life, that therefore he was thus healthy. Persons have lived in good health to the age of three-score years and ten, who rose early and rose late; who drank liquor three times a day, (or an indefinite number of times,) and did not drink it at all; who were out of doors a great deal, or seldom had the sunshine on them; who were very good, and who were very bad. The object of these statements is to show the fallacy of attributing a long and healthful life to one thing, to its presence or its absence, and to direct attention to this important truth, one which strikingly exhibits the wisdom and the love of our Father in heaven. That men can live long in any country, clime, or latitude, in the use of the things around them, by wisely adapting themselves to their circumstances, in temperance, industry and equanimity; that these not only of themselves promote length of days, but antagonize the baleful effects of deleterious agencies. If a man does bathe every day, or never uses tea, coffee, liquor, or tobacco, or eschews fish, flesh, and fowl, he will not be exempt from disease and premature death, unless he is temperate, careful, systematic, and serene, and with these, he can cover "a multitude of sins" physical.

More than a year ago, we met a shipping-merchant, an old friend, one of the most estimable of men as a husband, father, citizen, and merchant. He began to expatiate on the enjoyment of a regular morning bath; the delight, the recreation, the glow and subsequent feeling of refreshment. "I never take a cold, am regular as a clock, eat no suppers, take nothing from breakfast until five o'clock dinner, for which I have a relish which is delightful, and as I think, owing to my regular cold morning shower-bath."

"Were you in good health when you began?"

"Oh! yes, always had good health."

Three months ago we saw him at his own house; not the portly, vivacious, and rubicund man of the previous year; the cheek had faded, the flesh had flabbed, the eye had paled of its lustre, and the voice itself was subdued and sad. He was the victim of an agonizing chronic disease.

It should be the wisdom of all to look for a healthful life, not in this one thing, or that or the other; but in the cultivation of a habit of temperance, industry, and equanimity.

